# FKBP39 controls nutrient dependent Nprl3 expression and TORC1 activity in Drosophila

**DOI:** 10.1038/s41419-021-03860-z

**Published:** 2021-06-02

**Authors:** Ying Zhou, Jian Guo, Xinyu Wang, Yang Cheng, Jianwen Guan, Priyam Barman, Ming-An Sun, Yuanyuan Fu, Wanhong Wei, Congjing Feng, Mary A. Lilly, Youheng Wei

**Affiliations:** 1 Joint International Research Laboratory of Agriculture and Agri-Product Safety, the Ministry of Education of China, Yangzhou University, Yangzhou, 225009 China; 2 Animal Physiology Group, College of Bioscience and Biotechnology, Yangzhou University, Yangzhou, 225009 China; 3 Institute of Comparative Medicine, College of Veterinary Medicine, Yangzhou University, Yangzhou, 225009 China; 4 College of Horticulture and Plant Protection, Yangzhou University, Yangzhou, 225009 China; 5 Eunice Kennedy Shriver National Institute of Child Health and Human Development, National Institutes of Health, Bethesda, MD 20892 USA

**Keywords:** Autophagy, Proteasome, Proteasome, Metabolism

## Abstract

Target of Rapamycin Complex 1 (TORC1) is a master regulator that coordinates nutrient status with cell metabolism. The GTPase-activating protein towards Rags complex 1 (GATOR1) inhibits TORC1 activity and protects cells from damage during periods of stress. Here we characterize multiple pathways that regulate the expression of the GATOR1 component Nprl3 in Drosophila. We determine that the stability of Nprl3 is impacted by the Unassembled Soluble Complex Proteins Degradation (USPD) pathway. In addition, we find that FK506 binding protein 39 (FKBP39)-dependent proteolytic destruction maintains Nprl3 at low levels in nutrient replete conditions. Nutrient starvation abrogates the degradation of the Nprl3 protein and rapidly promotes Nprl3 accumulation. Consistent with a role in promoting the stability of a TORC1 inhibitor, mutations in *fkbp39* decrease TORC1 activity and increase autophagy. Finally, we show that the 5′UTR of *nprl3* transcripts contain a functional upstream open reading frame (uORF) that inhibits main ORF translation. In summary, our work has uncovered novel mechanisms of Nprl3 regulation and identifies an important role for FKBP39 in the control of cellular metabolism.

## Introduction

In eukaryotes, Target of Rapamycin Complex 1 (TORC1), an evolutionarily conserved serine/threonine kinase, coordinates nutrient and energy status with cell growth^1–3^. When nutrients are sufficient, TORC1 activity promotes protein synthesis by phosphorylating multiple substrates including, but not limited to, S6 kinases (S6Ks) and 4E-binding proteins (4E-BPs)^4^. S6Ks phosphorylate multiple translation factors, while 4E-BPs bind to and inhibit the cytoplasmic cap-binding subunit involved in translation initiation^5^.

Some stress signals, such as nutrient scarcity or growth factor shortage, reduce TORC1 activity and inhibit the initiation of cap-dependent translation^6,7^. Cap-dependent translation begins with the formation of the 43S preinitiation complex (PIC) that moves along the 5′ UTR and searches for an AUG start codon, where the 80S ribosome is formed to initiate translation until arriving at the stop codon for termination^8,9^. However, in eukaryotes, some transcripts contain upstream open reading frames (uORFs), short translated sequences defined by a start codon AUG and an in-frame stop codon, within their 5′UTR^10–12^. Ribosome profiling, a method based on deep sequencing of ribosome-protected mRNA fragments (RPFs), is widely used to identify functional uORFs, which appear in ribo-seq data together with the actively translated main ORF^13,14^. Typically, functional uORFs act as translation inhibitors of their downstream main ORF^8,10^.

Amino acids control TORC1 activity by modulating the guanine nucleotide-binding status of heterodimeric Rags GTPase RagA/B and RagC/D^15,16^. The Rag GTPase promotes TORC1 activity when RagA/B is bound to GTP and RagC/D is bound to GDP. Conversely, the Rag GTPase functions to inhibit TORC1 activity when RagA/B is in the GDP state and Rag C/D is GTP bound^17,18^. The GTPase-activating protein (GAP) towards Rags (GATOR) complex, comprised of two subcomplexes GATOR1 and GATOR2, mediate cellular amino acid levels and TORC1 activity^19^. The GATOR1 complex, composed of Nprl2, Nprl3 and Iml1/DEPDC5, displays GAP activity that hydrolyzes the RagA/B bound GTP to GDP and releases TORC1 from the lysosome to inhibit its activity^19^. The GATOR2 complex physically interacts with and inhibits the GATOR1 complex and functions to promote TORC1 activity^15,20^. Cytoplasmic amino acids control TORC1 activity through amino acid sensors that bind to GATOR1 or GATOR2. Upon amino acid starvation, Sestrin2 and CASTOR1 bind and antagonize GATOR2 function, whereas SAMTOR and KCSTOR bind and promote GATOR1 function^21–24^. Interestingly, although the GATOR and TORC1 are evolutionally conserved in eukaryotes^25^, some amino acid mediators are absent in invertebrates. For example, the KICSTOR and the CASTOR1 complex are not encoded in the genome of *Drosophila melanogaster* and *Caenorhabditis elegans*^21,23^.

In mammalian cells, amino acids control the protein stability of the GATOR1 component DEPDC5, the homolog of Iml1^26^. When nutrients are sufficient, CUL3–KLHL22 E3 ligase promotes the ubiquitination and degradation of the DEPDC5 via the proteasome. Upon amino acid starvation, KLHL22 is bound and trapped by 14-3-3 proteins in the nucleus, resulting in a dramatic increase in DEPDC5 protein stability^26^. Interestingly, depletion of DEPDC5 results in the decreased expression of the other two GATOR1 components NPRL2 and NPRL3^23,27^. We reasoned that under amino acid starvation the expression levels of Nprl2 and Nprl3 might also be regulated, especially in the invertebrates that have no orthologs of KICSTOR and CASTOR1.

Here we identified multiple pathways that control Drosophila Nprl3 expression. We found that amino acid starvation enhanced Nprl3 expression, by preventing the FKBP39 dependent destruction of the Nprl3 protein via the proteasome. In addition, we showed that the Nprl3 protein is unstable in the absence of the other GATOR1 components, Nprl2 and Iml1. Finally, we determined that the uORF in the 5’UTR of the *nprl3* transcript is a functional *cis*-element that acts to reduce Nprl3 translation. Our data support the model that the regulation of Nprl3 expression functions as a key regulator of GATOR1 activity.

## Materials and methods

### Drosophila strains

The stocks *fkbp39 RNAi* (THU#0803), *huwe1 RNAi* (THU#1805) were obtained from Tsinghua Fly Center. The *Tsc1 RNAi* (BDSC#35144), *MTD-Gal4* (BDSC#31777) stocks were obtained from the Bloomington Drosophila Stock Center. The *Tub-GFP-LAMP1* was provided by Helmut Kramer (UT Southwestern)^28^. The *Nos-Gal4* was provided by Sharon Bickel (Dartmouth College)^29^. The *nprl2 RNAi*, *nprl3 RNAi*, *UAS-HA-Nprl2*, *UAS-HA-Nprl3*, *UAS-V5-Nprl3*, *nprl2*^*1*^, *nprl3*^*1*^ were described previously^30–32^. All fly stocks were maintained on BDSC standard cornmeal medium at 25 °C.

### Cell culture and amino acid starvation

Drosophila S2 cells (Thermo Fisher, #R69007) were cultured in *Schneider’s* Drosophila medium (Merck, #S9895) supplemented with 10% fetal bovine serum (FBS, Thermo Fisher, #A3160802). Amino acid starvation medium was prepared based on the formulation of *Schneider’s* medium without the indicated amino acids. Specifically, the amino acid starvation media contain CaCl_2_ (5.40 mM), MgSO_4_ (15.06 mM), KCl (21.33 mM), KH_2_PO4 (3.31 mM), NaHCO_3_ (4.76 mM), NaCl (36.21 mM), Na_2_HPO_4_ (4.94 mM), Alpha-Ketoglutaric acid (1.37 mM), D-Glucose (11.11 mM), Fumaric acid (0.86 mM), Malic acid (0.75 mM), Succinic acid (0.85 mM), Trehalose (5.85 mM). For amino acid starvation, cultured S2 cells or dissected Drosophila ovaries were incubated in the amino acid starvation media for the indicated time.

### Plasmids and cell transfection

The plasmids were constructed using a recombination kit according to the manufacturer’s instruction (Vazyme, #C112-01). The *nprl3*, *GFP*, and *FKBP39* coding regions were amplified using specific primers and then inserted into a pAC5.1-His-V5 vector (Invitrogen, #V411020) to construct the pAct-Nprl3-His-V5, pAct-GFP-His-V5, and pAct-FKBP39-His-V5 plasmids. The 5′UTRs and 3′UTR of *nprl3* were amplified using specific primers and inserted into pAct-GFP-His-V5 to construct a series of GFP reporter plasmids. The 5′UTRs, 3′UTR, and mutated 5′UTRs of *nprl3* were amplified using specific primers and inserted into psiCKECK2 (Promega, #C8021) to construct a series of Renilla luciferase reporter plasmids. The *nprl3* and *FKBP39* coding regions were amplified using specific primers and then inserted into a pENTR-1A vector (Invitrogen, #A10462). The pENTR-Nprl3 and pENTR-FKBP39 plasmids were recombined into pAHW (DGRC) to generate pAct-HA-Npr3 and pAct-HA-FKBP39 plasmids using Gateway LR Clonase II Enzyme (Invitrogen, #11791020). Primer sequences used to generate plasmids are listed in Supplementary Table 3. S2 cells grown in six-well plates were transfected with 0.2 μg plasmids using Effectene Transfection Reagent according to the manufacturer’s instruction (Qiagen, #301427).

### dsRNA and RNAi

To generate double-stranded RNA(dsRNA), DNA templates were PCR amplified to include a 5′ T7 RNA polymerase binding site. PCR products were used as templates to produce dsRNA using the MEGAscript RNAi kit according to the manufacturer’s instruction (Invitrogen, #AM1626). Primer sequences used to generate DNA templates for synthesizing double-strand RNA (dsRNA) and plasmids are listed in Supplementary Table 3. For RNAi, S2 cells were incubated with 15 μg dsRNA in six-well plates contained 0.5 mL Schneider’s medium without FBS for 1 h, and then 1.5 mL Schneider’s medium supplemented with 10% FBS was added and incubated for 3 days.

### Tandem affinity purification and mass spectrometry

Ovaries from the transgenic flies that stably express HA-FLAG tagged Nprl3 proteins and from control *yw* flies were homogenized and perform analysis as previously described^30^.

### Immunoprecipitation (IP) and western blot analysis

For IPs, the S2 cells transfected with specific plasmids were lysed using 1 ml RIPA buffer (Merck, #20-188) containing complete protease inhibitor (Roche, #1697498001) and phosphatase inhibitor (Roche, #4906845001). The protein extracts were incubated with 30 μL HA antibody-coated magnet beads for 6 h at room temperature. The beads were collected and washed six times with lysis buffer, and then detected by western blot. For western blot, antibodies were used at the following concentrations for western blot: mouse anti-α-tubulin at 1:10,000 (Beyotime, #AF0001), mouse anti-V5 antibody at 1:4000 (Invitrogen, # R960-25), rabbit anti-pS6K at 1:1000 (Cell Signaling Technology, #9209), rabbit anti-HA antibody at 1:1000 (Beyotime, #AF0039), guinea pig anti-Nprl3 (generated at this work) at 1:2000.

### RNA isolation and mRNA analysis

RNA isolation and qRT-PCR were performed as previously described^33^ using the primers list in Supplemental Table.

### Luciferase reporter assay

S2 cells transfected with pSiCHECK-2 plasmids that contained 5′UTR or 3′UTR of *nprl3* transcripts were lysed and the firefly luciferase (FL) and Renilla luciferase (RL) activities were measured using a dual luciferase assay system kit according to the manufacturer’s instruction (Promega, #E1910). The RL activity was normalized to the activity of FL.

### CHX chase studies for protein stability measurement

For protein stability assays, S2 cells were incubated with 10 μg/ml cycloheximide (CHX, Millipore, #66819) to inhibit mRNA translation. At the indicated time points after the addition of CHX, cells were harvested and the protein expression was detected using western blot. The α-Tubulin protein levels were used for normalization. The remaining protein was determined by comparison with the expression level at the time point when CHX was added.

### Generation of *fkbp39* mutant lines

*fkbp39* mutant fly was generated similarly as described previously^32^. Primers used for cloning and detection are listed in Supplemental Table 1.

### LysoTracker staining

The Drosophila ovaries were dissected in *Schneider’s* medium supplemental with 10% FBS. The tissues were incubated with LysoTracker Red (1:2000, Thermo Fisher, #L7528) and Hoechst (1:10,000, Thermo Fisher, #62245) for 5 min, and then washed and mounted using Schneider’s medium supplemental with 10% FBS. Images were acquired using a Zeiss LSM 880 confocal microscope.

### Ribosome profiling data analysis

Ribo-Seq data from the previous study were retrieved from NCBI Sequence Read Archive database under accession SRP067542^13^. The reads were trimmed with Trim Galore (https://www.bioinformatics.babraham.ac.uk/projects/trim_galore/) to remove adapter sequences and bad quality bases, and then aligned to D. melanogaster reference genome (release dm6) using STAR with default settings^34^. After excluding reads aligned to rRNAs, tRNAs, and snoRNAs, remaining aligned reads between 27 and 34 nt were considered as RPFs for further analysis. Using the count function of igv tools, the alignment files were converted to TDF format for visualization in IGV^35^.

### Statistical analyses

Data are reported as mean ± SD of at least three independent experiments and analyzed using a two-tailed *t*-test.

## Results

### Nprl3 protein stability is regulated by the unassembled soluble complex protein degradation (USPD) pathway

**Fig. 1:**
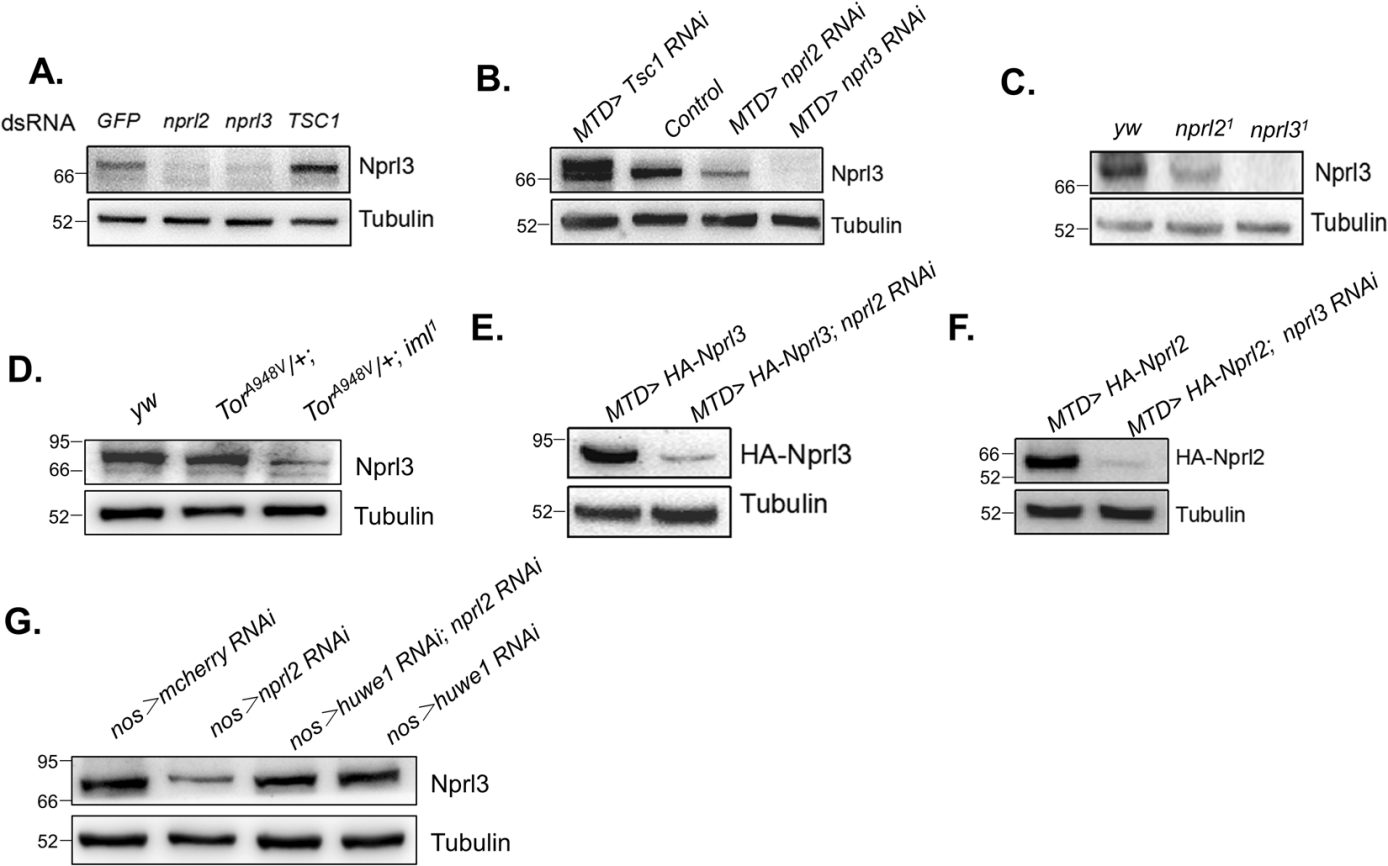
Nprl3 and Nprl2 expression levels are interdependent. **A** Western blot analysis of Nprl3 protein in the indicated dsRNA treated S2 cells. **B** Western blot analysis of Nprl3 protein in the ovaries of *MTD* > *mCherry RNAi* (control), *MTD* > *nprl2 RNAi*, *MTD* > *nprl3 RNAi* and *MTD* > *Tsc1 RNAi* flies. **C** Western blot analysis of Nprl3 protein in the ovaries of *yw* (control), *nprl2*^*1*^ and *nprl3*^*1*^. **D** Western blot analysis of Nprl3 protein in the ovaries of *yw*, *Tor*^*A948V*^*/+* and *Tor*^*A948V*^*/+; iml1*^*1*^. **E** Western blot analysis of HA-Nprl3 protein in the ovaries of *MTD* > *HA-Nprl3* and *MTD* > *HA-Nprl3, nprl2 RNAi* flies. **F** Western blot analysis of the HA-Nprl2 protein in the ovaries of *MTD* > *HA-Nprl2* and *MTD* > *HA-Nprl2, nprl3 RNAi* flies. **G** Western blot analysis of Nprl3 protein in the ovaries of *nos* > *mCherry RNAi* (control)*, nos* > *nprl2 RNAi*, *nos* > *nprl2 RNAi, huwe1 RNAi* and *nos* > *huwe1 RNAi* flies. α-Tubulin was used as a loading control. Similar western blot results were observed in more than three independent experiments for each group.

GATOR1 promotes the conversion of RagA/B-GTP to RagA/B- GDP, which results in the release of TORC1 from lysosomes and an accompanying loss of TORC1 activity^19^. To detect endogenous GATOR1 expression, we generated a guinea pig anti-Nprl3 antibody that specifically recognizes Drosophila Nprl3 (Supplemental Fig. 1). Interestingly, in mammalian cells mutations in the GATOR1 component *DEPDC5* decrease the expression of the other two GATOR1 components Nprl2 and Nprl3^23,27^. Consistent with this interdependence, we determined that knocking down *nprl2* decreased Nprl3 expression in Drosophila S2 cells (Fig. 1A). Similarly, the levels of Nprl3 protein were decreased in the ovaries of *nprl2* mutants or in the ovaries expressing an *nprl2* RNAi using a germline-specific MTD-GAL4 driver (Fig. 1B, C). In contrast, knocking down *Tsc1*, an independent inhibitor of TORC1, did not decrease Nprl3 expression, which suggested that the decreased Nprl3 expression was not caused by TORC1 hyperactivation (Fig. 1A, B). Next, we detected the levels of Nprl3 protein in the mutant of *iml1*, homologue of DEPDC5 in Drosophila. Consistently, the levels of Nprl3 protein were decreased in the ovaries of *iml1*^*1*^ mutant with *Tor*^*A948V*^ heterozygous, which rescued the *iml1*^*1*^ lethality^32^ (Fig. 1D). In eukaryotes, the USPD pathway is thought to prevent protein aggregation^36,37^. Thus, we reasoned that the decreased expression of Nprl3 in *nprl2* depletion cells might reflect the activation of the USPD pathway. To test this model, we detected the expression of exogenous HA-tagged Nprl3 and found that *nprl2* depletions decreased the levels of ectopically expressed HA-Nprl3 protein in the Drosophila ovary (Fig. 1E). Conversely, knocking down *nprl3* transcript levels decreased HA-tagged Nprl2 expression in the Drosophila ovary (Fig. 1F). Recently, the HECT, UBA and WWE domain-containing E3 ubiquitin-protein ligase 1 (HUWE1) was reported to interact with many multi-protein complexes to promote the USPD process^36^. Interestingly, depletion of HUWE1 rescued the expression defect of Nprl3 in the Drosophila *nprl2* RNAi ovaries (Fig. 1G). These data strongly suggest that the expression of the GATOR1 components are interdependent and controlled by the USPD pathway.

### Amino acid starvation increases Nprl3 protein levels

**Fig. 2:**
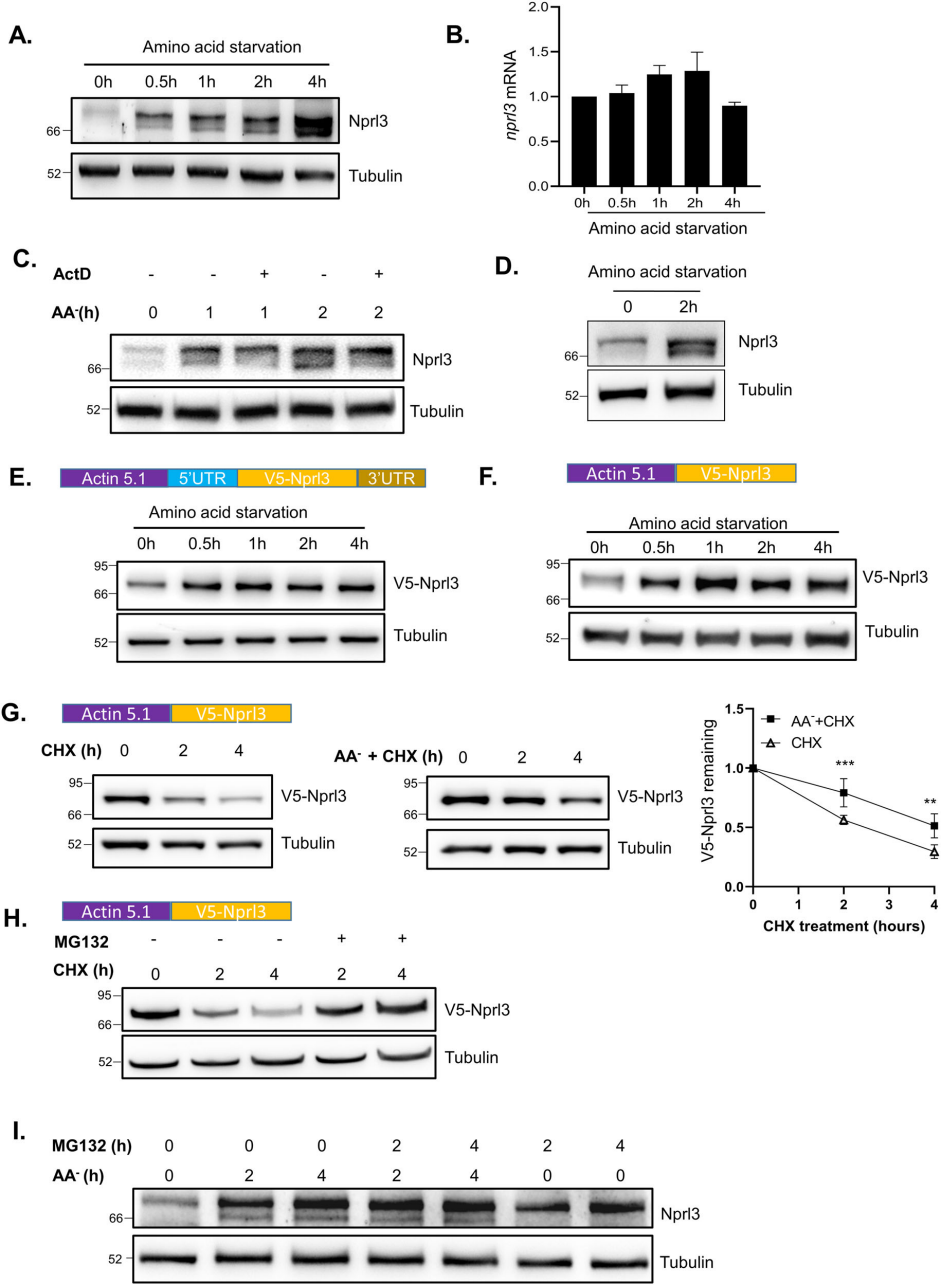
Amino acid starvation enhances Nprl3 protein stability. **A** Western blot analysis of Nprl3 protein in S2 cells under amino acid starvation. **B** qRT-PCR analysis of *nprl3* mRNA in S2 cells under amino acid starvation. Error bars represent standard error values from three independent experiments. **C** Western blot analysis of the Nprl3 protein in S2 cells treated with actinomycin D together with amino acid starvation. **D** Ovaries from *yw* flies were dissected and cultured in amino-acid-free Schneider’s medium (AA-) for 2 h. Nprl3 was detected using Western blot. **E** S2 cells were transfected with the pAC5.1 construct inserted with *nprl3* 5’UTR, V5-tagged Nprl3 coding region, and *nprl3* 3′UTR. The cells were starved for the indicated time and the V5-Nprl3 levels were measured by western blot. **F** S2 cells were transfected with the pAC5.1 construct inserted with V5-tagged Nprl3 coding region. The cells were starved for the indicated time and the V5-Nprl3 levels were measured by western blot. **G** S2 cells were transfected with the pAC5.1 construct inserted with V5-tagged Nprl3 coding region. The cells were treated with CHX alone or CHX together with amino acid starvation medium for the indicated time. The V5-Nprl3 levels were measured by western blot. The remaining protein levels at each time point were calculated, taken the expression level of V5-Nprl3 at 0 h as 1. Error bars represent standard error values from three independent experiments. **P < 0.01; ***P < 0.001. **H** S2 cells were transfected with the pAC5.1 construct inserted with V5-tagged Nprl3 coding region. The cells were treated with CHX alone or CHX together with MG132 for the indicated time. The V5-Nprl3 levels were measured by western blot. **I** Western blot analysis of endogenous Nprl3 protein in S2 cells under amino acid starvation and MG132 treatment. *Rp49* was used for normalization for qPCR. α-Tubulin was used as a loading control for western blot. Similar western blot results were observed in more than three independent experiments for each group.

Previously, we demonstrated that the GATOR1 complex is required for Drosophila viability and young egg chamber survival under nutrient stress conditions^31,32^. Based on our findings that the expression of GATOR1 components are interdependent and that DEPDC5/Iml1 expression is sensitive to nutrient status in mammals^26^, we reasoned that the levels of the GATOR1 component Nprl3 might also be sensitive to amino acid starvation. First, we examined whether the expression of Nprl3 is affected by nutrient status in Drosophila. Interestingly, amino acid starvation significantly increased the expression of Nprl3 protein in Drosophila S2 cells (Fig. 2A). By contrast, exposure of S2 cells to amino acid starvation did not change *nprl3* mRNA levels significantly, as assessed by RT-qPCR amplification (Fig. 2B). In addition, actinomycin D, an inhibitor of transcription, did not prevent the increase in Nprl3 levels observed under amino acid starvation (Fig. 2C). The Drosophila ovary has a high level of Nprl3 expression (Supplemental Fig. 2). To determine whether Nprl3 protein level is induced by nutrient depletion in vivo, we dissected and incubated Drosophila ovaries in amino acid starvation media for 2 h and found that Nprl3 levels increased significantly in dissected ovaries after amino acid starvation (Fig. 2D). Consistent with these observations, when we incubated female flies in 20% sucrose media with no amino acids for 1 day, we found that Nprl3 protein levels significantly increased in ovaries (Supplemental Fig. 3). To confirm that the increased Nprl3 expression observed under amino acid starvation is independent of transcription, we constructed a plasmid that contains the 5’UTR, V5-tagged coding region, and 3’UTR of the *nprl3* mRNA under the control of the actin promoter in pAC5.1 vector. We found that even when Nprl3 was expressed from the actin promoter, amino acid starvation significantly increased V5-tagged Nprl3 protein levels in S2 cells (Fig. 2E). Thus, the increase in Nprl3 protein expression upon amino acid starvation is driven by a posttranscriptional mechanism. Taken together our data indicate that the increase in Nprl3 protein expression observed upon amino acid starvation in both S2 cells and the Drosophila ovary is driven by a posttranscriptional mechanism.

Next, we wanted to determine if the increased Nprl3 protein observed upon amino acid starvation mapped to the coding region of Nprl3, suggesting a possible change in protein stability. To answer this question, we transfected S2 cells with a construct that contained the V5-tagged Nprl3 coding region but not the 3′ or 5′ UTRs and found that amino acid starvation still induced increased V5-Nprl3 levels in starved cells (Fig. 2F). These data are consistent with the idea that nutrient starvation impacts the stability of the Nprl3 protein. To test this model, we examined Nprl3 protein stability in the presence of the translational inhibitor cycloheximide (CHX). Because the endogenous Nprl3 protein is too low under amino acid sufficient condition to detect in S2 cells, we followed the ectopically expressed V5-tagged Nprl3 protein. Notably, amino acid starvation significantly decreased the degradation rate of V5-Nprl3 protein (Fig. 2G). To determine whether Nprl3 protein degradation is mediated by the proteasome, we used MG132 to inhibit the proteolytic activity of the 26S proteasome complex. We found that MG132 treatment significantly inhibited V5-Nprl3 protein degradation (Fig. 2H). Moreover, similar to amino acid starvation treatment, MG132 treatment increased endogenous Nprl3 levels in non-starvation conditions (Fig. 2I). Importantly, subjecting cells to MG132 treatment and amino acid starvation did not significantly increase the levels of Nprl3 protein above either single treatment (Fig. 2I). These data suggest that MG132 treatment and amino acid starvation likely impact the same pathway. Taken together our data strongly suggest that amino acid starvation increases Nprl3 protein stability at least in part by inhibiting proteasome-dependent degradation in nutrient replete conditions.

### FKBP39 associates with Nprl3 and regulates its stability

**Fig. 3:**
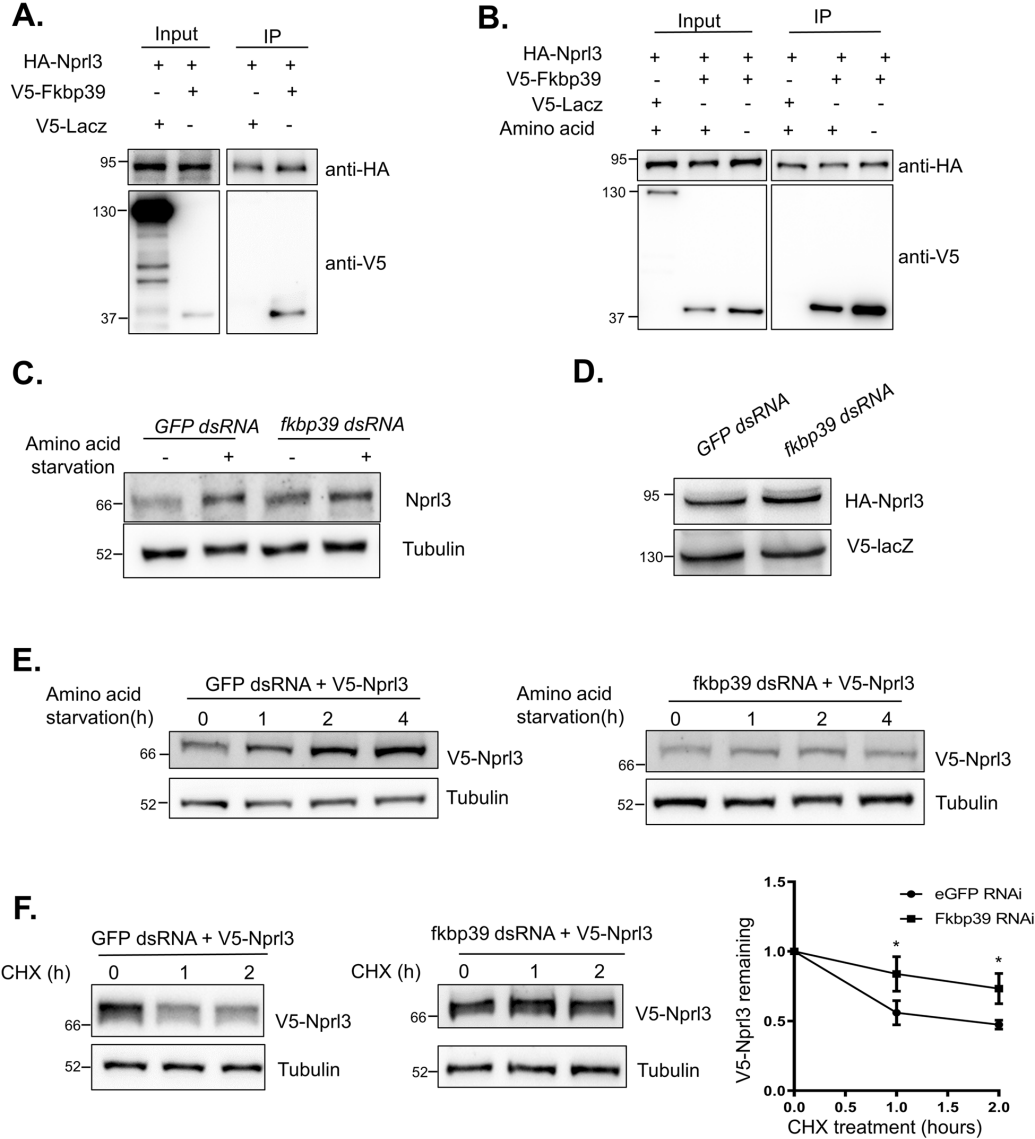
FKBP39 associates with Nprl3 and promotes Nprl3 degradation. **A** S2 cells were co-transfected with HA-tagged Nprl3 and V5-tagged FKBP39 or lacZ (control) plasmids. Cells were lysed and immunoprecipitated using beads coated with anti-HA antibody. Cell lysates (inputs) and immunoprecipitates (IP) were detected by western blot. **B** S2 cells were co-transfected with HA-tagged Nprl3 and V5-tagged FKBP39 or lacZ (control) plasmids. Cells were cultured in Schneider’s medium plus 10% FBS (AA+) or amino-acid-free Schneider’s medium (AA−) for 2 h. The cells were lysed and immunoprecipitated using beads coated with an anti-HA antibody. Cell lysates (inputs) and immunoprecipitates (IP) were detected by western blot. **C** S2 cells were transfected with *GFP* (control) or *fkbp39* dsRNA. Cells were treated with amino acid starvation for 2 h. The levels of Nprl3 were detected by western blot. **D** S2 cells were co-transfected with the *GFP* (control) or *fkbp39* dsRNA, constructs expressed HA-tagged Nprl3 and V5 tagged lacZ. The levels of HA-Nprl3 and V5-lacZ were detected by western blot. V5-lacZ was used as a loading control for western blot. **E** S2 cells were co-transfected with the *GFP* (control) or *fkbp39* dsRNA and construct expressed V5-tagged Nprl3. Cells were treated with amino acid starvation for the indicated hours. The levels of V5-Nprl3 were detected by western blot. **F** S2 cells were co-transfected with the *GFP* (control) or *fkbp39* dsRNA and construct expressed V5-tagged Nprl3. The cells were treated with CHX for the indicated time and the V5-Nprl3 levels were measured by western blot. The remaining protein levels at each time point were calculated, taken the expression level of V5-Nprl3 at 0 h as 1. Error bars represent standard error values from three independent experiments. *P < 0.05. α-Tubulin was used as a loading control for western blot. Similar western blot results were observed in more than three independent experiments for each group.

To elucidate mechanistically why Nprl3 stability increases upon amino acid starvation, we used immunoprecipitation followed by quantitative mass spectrometry to identify proteins that physically interact with the Nprl3 protein. Immunoprecipitations were performed using lysates generated from control and HA-FLAG-Nprl3 expressing (*MTD-GAL4* driver) Drosophila ovaries. The immunoprecipitates were subject to mass spectrometry analysis for quantitation. As anticipated, additional GATOR complex components including Nprl2, Iml1, Seh1, and Mio were specifically pulled down from the HA-FLAG-Nprl3 lysate (Supplemental Table 1). Notably, we found that the FK506 binding protein 39 KD (FKBP39) was also enriched in the HA-FLAG-Nprl3 ovary lysate. To verify the interaction between Nprl3 and FKBP39, we co-expressed HA-tagged Nprl3 and V5-tagged FKBP39 in Drosophila S2 cells and carried out co-immunoprecipitation assays using HA antibody-coated beads. The immunoprecipitation of HA-tagged Nprl3 co-precipitated V5-tagged FKBP39, but not the negative control V5-tagged LacZ (Fig. 3A). Next, we detected whether the interaction is affected by nutrient status. Amino acid starvation did not significantly affect the physical interaction between FKBP39 and Nprl3 in S2 cells, suggesting the association between these two proteins is amino acid insensitive (Fig. 3B).

To evaluate the effect of FKBP39 on Nprl3 protein levels, the endogenous Nprl3 protein in *fkbp39* RNAi cells was detected using western blot. Knocking down FKBP39 increased Nprl3 levels and these levels could not be increased further by amino acid starvation (Fig. 3C). We speculated that the FKBP39 protein destabilizes the Nprl3 protein under amino acid sufficient condition and this destabilization is alleviated by amino acid starvation. To test this hypothesis, the ectopically expressed HA-tagged Nprl3 protein was detected in *fkbp39* RNAi cells. Compared to the control cells, the expression level of HA-Nprl3 protein was much higher in *fkbp39* RNAi cells (Fig. 3D). Moreover, amino acid starvation could not increase the levels of ectopically expressed V5-Nprl3 in *fkbp39* RNAi cells (Fig. 3E). Next, we detected the effect of FKBP39 on the stability of Nprl3 protein and found that the decay rate of the V5-tagged Nprl3 protein was much slower in *fkbp39* RNAi cells (Fig. 3F). In summary, our data strongly suggest that FKBP39 associates with the Nprl3 protein and promotes its degradation in nutrient-replete conditions.

### FKBP39 associates with Nprl2 and regulates its expression

**Fig. 4:**
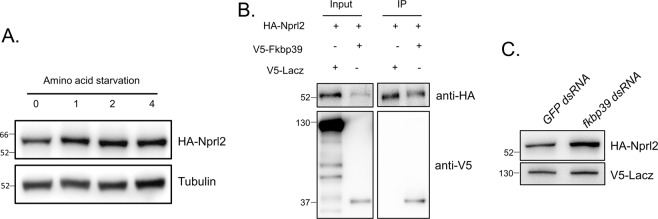
FKBP39 associates with Nprl2 and decreases its expression. **A** S2 cells were transfected with the indicated construct inserted with HA-tagged Nprl2 coding region. The cells were starved for the indicated time and the HA-Nprl2 levels were measured by western blot. α-Tubulin was used as a loading control for western blot. **B** S2 cells were co-transfected with HA-tagged Nprl2 and V5-tagged FKBP39 or lacZ (control) plasmids. Cells were lysed and immunoprecipitated using beads coated with anti-HA antibody. Cell lysates (inputs) and immunoprecipitates (IP) were detected by western blot. **C** S2 cells were co-transfected with the *GFP* (control) or *fkbp39* dsRNA, constructs expressed HA-tagged Nprl2 and V5-tagged lacZ. The levels of HA-Nprl2 and V5-lacZ were detected by western blot. V5-lacZ was used as a loading control for western blot. Similar western blot results were observed in more than three independent experiments for each group.

Next, we determine whether amino acid starvation and FKBP39 regulates the expression levels of another GATOR1 component, Nprl2 in Drosophila. Due to the lack of a suitable anti-Nprl2 antibody, we measured the expression level of ectopically expressed HA-tagged Nprl2. Interestingly, the levels of HA-Nprl2 increased upon amino acid starvation (Fig. 4A). We co-expressed HA-tagged Nprl2 and V5-tagged FKBP in Drosophila S2 cells and carried out co-immunoprecipitation assays using HA antibody-coated beads. The immunoprecipitation of HA-tagged Nprl2 co-precipitated V5-tagged FKBP39, but not the negative control V5-tagged LacZ (Fig. 4B). Next, we examined the effect of FKBP39 on Nprl2 expression. Compared to control cells, the expression level of HA-Nprl2 protein was much higher in *fkbp39* RNAi cells (Fig. 4C). These results indicate that FKBP39 regulates the stability of multiple GATOR1 components.

### FKBP39 promotes TORC1 activity in vivo

**Fig. 5:**
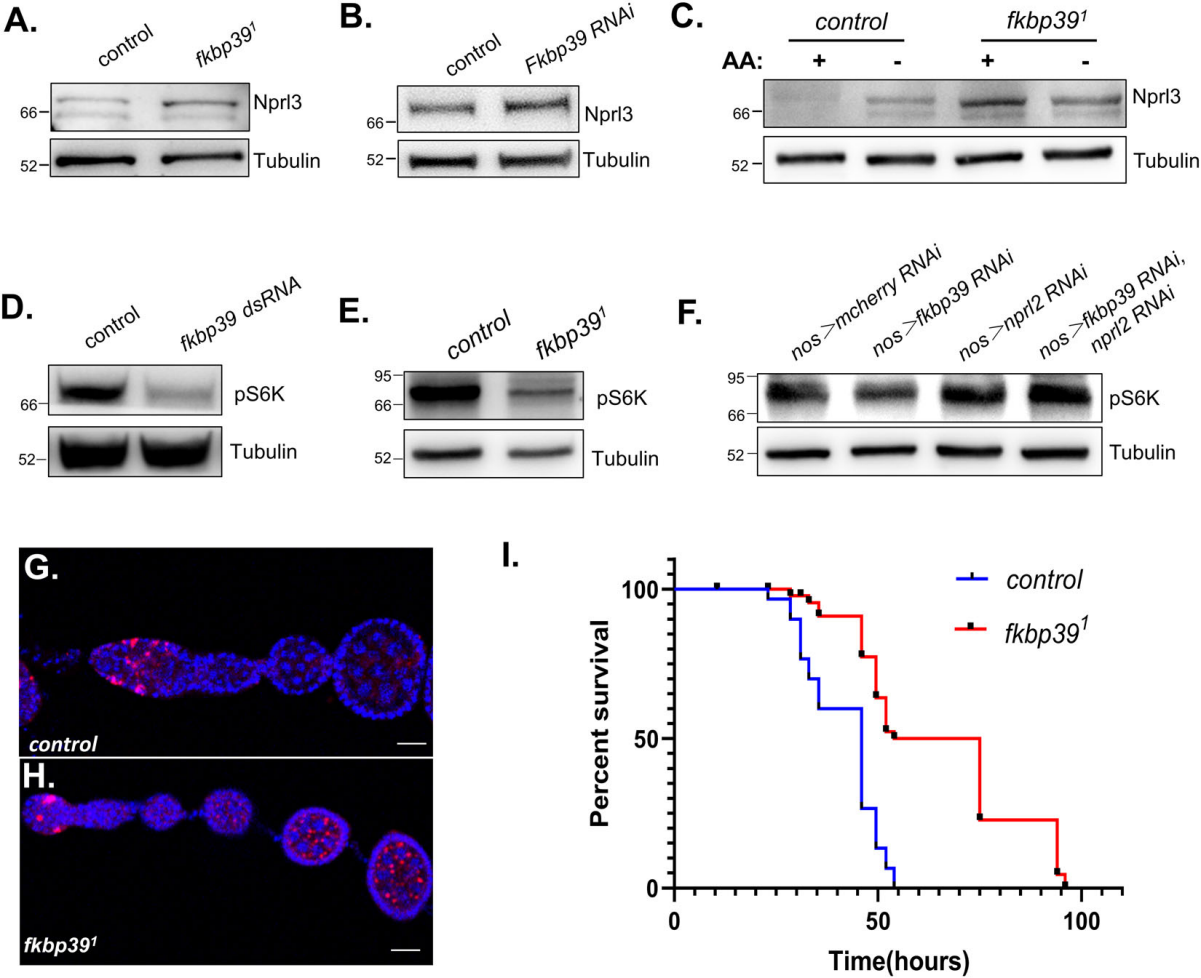
FKBP39 increases TORC1 activity in vivo. **A** Western blot analysis of Nprl3 protein in the ovaries of *yw* (control) and *fkbp39*^*1*^. **B** Western blot analysis of the Nprl3 protein expression in the ovaries of *MTD* > *mCherry RNAi* (control) and MTD > *fkbp39 RNAi* flies. **C** The ovaries from *yw* (control) and *fkbp39*^*1*^ were dissected and cultured in amino-acid-free Schneider’s medium (AA-) for 2 h. The levels of Nprl3 were detected by western blot. **D** Western blot analysis of phosphorylated S6K in the *GFP* (control) or *fkbp39* dsRNA treated S2 cells. **E** Western blot analysis of phosphorylated S6K in the ovaries of *yw* (control) and *fkbp39*^*1*^. **F** Western blot analysis of phosphorylated S6K in the ovaries of *nos* > *mCherry RNAi* (control), *nos* > *fkbp39 RNAi, nos* > *nprl2 RNAi* and *nos* > *fkbp39 RNAi, nprl2 RNAi* flies. **G**–**H** The ovaries from *yw* (control) and *fkbp39*^*1*^ were dissected in cell culture media and stained with Hoechst and LysoTracker Red. Bar, 20 μm. **I** Three days old *yw* (*n* = 30) and *fkbp39*^*1*^ (*n* = 44) flies were cultured with complete starvation media (1% Agar in PBS). Survivors were counted at the indicated time points. Pairwise comparisons by the Mantel-Cox log rank test showed *P* < 0.001. α-Tubulin was used as a loading control for western blot. Similar western blot results were observed in more than three independent experiments for each group.

To confirm the function of FKBP39 in regulating Nprl3 protein expression in vivo, we generated *fkbp39* mutant flies, in which the coding region of the *fkbp39* gene was removed using the CRISPR/Cas9 method. The mutant, named *fkbp39*^*1*^, has more than 90% deletion in the coding region (Supplemental Fig. 4). Interestingly, *fkbp39*^*1*^ was viable and had no obvious defects. We detected the Nprl3 expression in *fkbp39* mutant and RNAi flies. The expression level of Nprl3 protein in *fkbp39* mutant or *fkbp39* RNAi ovaries was much higher than that in control (Fig. 5A, B). Next, we dissected ovaries from wild-type or *fkbp39*^*1*^ females and cultured them in amino acid starvation media. The expression level of Nprl3 in *fkbp39*^*1*^ ovary was higher than control and could not be increased by amino acid starvation (Fig. 5C). To determine there are stage-specific differences in the accumulation of Nprl3 protein during oogenesis, we examined the distribution of Nprl3 in the Drosophila ovary using immunofluorescence. As we previously reported, Nprl3 localizes to lysosomes and autolysosomes that appear as bright puncta in the cytoplasm^31^. We found that both starvation and *fkbp39* mutation increased Nprl3 puncta brightness mainly in the germanium and young egg chambers (supplemental Fig. 5). In summary, these results indicate that FKBP39 inhibits Nprl3 accumulation and this effect can be alleviated by nutrient starvation in vivo.

As Nprl3 is an inhibitor of TORC1, FKBP39 might promote TORC1 activity through decreasing Nprl3 levels. We assayed TORC1 activity by measuring the levels of phosphorylated S6K, a downstream effector of TORC1. Notably, the TORC1 activity significantly decreased in FKBP39 knockdown S2 cells and *fkbp39* mutant ovaries (Fig. 5D, E). To confirm the effect of FKBP39 on TORC1 is mediated by the GATOR1 complex, we detected the phosphorylated S6K in ovaries with germline knockdowns of both FKBP39 and Nprl2. As expected, the effect of FKBP39 knockdown on decreasing TORC1 activity was eliminated by depletion of GATOR1 component Nprl2 (Fig. 5F). These results strongly suggest that FKBP39 maintains TORC1 activity by decreasing GATOR1 expression.

As a master mediator of nutrient status and cell metabolism, TORC1 controls cell growth and autophagy. Autophagy is a catabolic process that uses lysosomal degradation to eliminate damaged proteins and provide nutrients for cell survival under stress conditions^38^. Nutrient starvation induces autophagy through inhibition of TORC1^39^. To confirm the decreased TORC1 activity in *fkbp39* mutant, we stained the Drosophila ovaries with LysoTracker, which has been commonly used to examine autophagy for it stains acidic lysosomes and autolysosomes^40^. In fed conditions, the young egg chambers from the wild-type flies had few LysoTracker-positive puncta (Fig. 5G). In contrast, the egg chambers from *fkbp39* mutant flies contained more and larger LysoTracker-positive puncta (Fig. 5H). LAMP1 is a widely used lysosome and autolysosome marker for autophagy detection^40^. Consistent with the LysoTracker staining, the GFP–LAMP1 positive puncta in *fkbp39* mutants were more and larger than in control flies (Supplemental Fig. 6). Decreased TORC1 promotes lifespan and starvation resistance in Drosophila^41^. Interestingly, *fkbp39* mutant had increased longevity under starvation, consistent with the low TORC1 activity in *fkbp39* mutant animals (Fig. 5I). These results indicate that FKBP39 promotes TORC1 activity and regulates cell metabolism in vivo.

### Nutrient status and the USPD pathway independently control Nprl3 stability

**Fig. 6:**
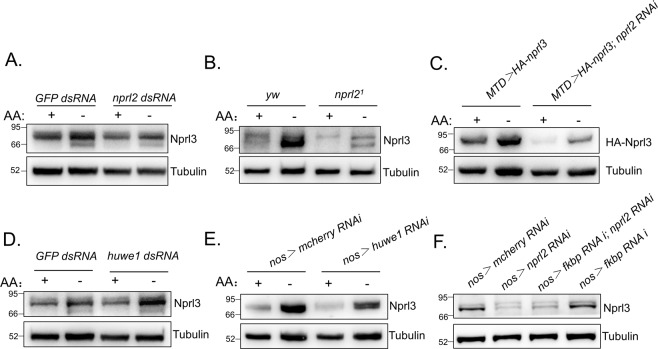
Nutrient signal and USPD independently controls Nprl3 stability. **A** S2 cells were transfected with *GFP* (control) or *nprl2* dsRNA. Cells were treated with amino acid starvation for 2 h. The levels of Nprl3 were detected by western blot. **B** The ovaries from *yw* (control) and *nprl2*^*1*^ were dissected and cultured in amino-acid-free Schneider’s medium (AA−) for 2 h. The levels of Nprl3 were detected by western blot. **C**. The ovaries from *MTD* > *Nprl3-HA* (control) and *MTD* > *Nprl3-HA, nprl2 RNAi* were dissected and cultured in amino-acid-free Schneider’s medium (AA−) for 2 h. The levels of HA-Nprl3 were detected by western blot. **D** S2 cells were transfected with *GFP* (control) or *huwe1* dsRNA. Cells were treated with amino acid starvation for 2 h. The levels of Nprl3 were detected by western blot. **E** The ovaries from *nos* > *mCherry RNAi* (control) and *nos* > *huwe1 RNAi* were dissected and cultured in amino-acid-free Schneider’s medium (AA-) for 2 h. The levels of Nprl3 were detected by western blot. **F** Western blot analysis of Nprl3 protein in the ovaries of *Nos* > *mCherry RNAi* (control), *Nos* > *nprl2 RNAi, Nos* > *nprl2 RNAi, fkbp39 RNAi* and *Nos* > *fkbp39 RNAi* flies. α-Tubulin was used as a loading control. Similar western blot results were observed in more than three independent experiments for each group.

Both nutrient status and the USPD pathway controls the stability of GATOR1 components. To address whether the regulation of Nprl3 expression by nutrient signals is dependent on the USPD pathway, we examined the changes of Nprl3 expression upon amino acid starvation in *nprl2* RNAi cells by western blot. Interestingly, amino acid starvation still significantly increased Nprl3 levels in *nprl2* RNAi S2 cells (Fig. 6A). In addition, amino acid starvation increased Nprl3 levels in *nprl2* mutant ovaries (Fig. 6B). Finally, amino acid starvation increased exogenous HA-tagged Nprl3 levels in *nprl2* RNAi ovaries (Fig. 6C). Next, we examined Nprl3 expression in cells and animals in which the USPD component HUWE1 was depleted. Consistent with the above results, amino acid starvation increased Nprl3 levels in HUWE1 RNAi cells both in vitro and in vivo (Fig. 6D, E). These results strongly suggest that nutrient status controls Nprl3 expression independent of the USPD pathway. To determine whether FKBP39 acts in the USPD to regulate the levels of GATOR1 complex components, we detected Nprl3 expression in *nprl2* and *FKBP39* double knockdown cells. Unlike HUWE1 depletion, FKBP39 depletion could not rescue Nprl3 levels in *nprl2* RNAi cells (Fig. 6F). This result suggests that nutrient signal machinery component FKBP39 has no effect on USPD of the GATOR1 complex. In summary, our results suggest that nutrient signaling and the USPD pathway independently control GATOR1 expression.

### The *nprl*3 5′UTR contains functional uORFs

**Fig. 7:**
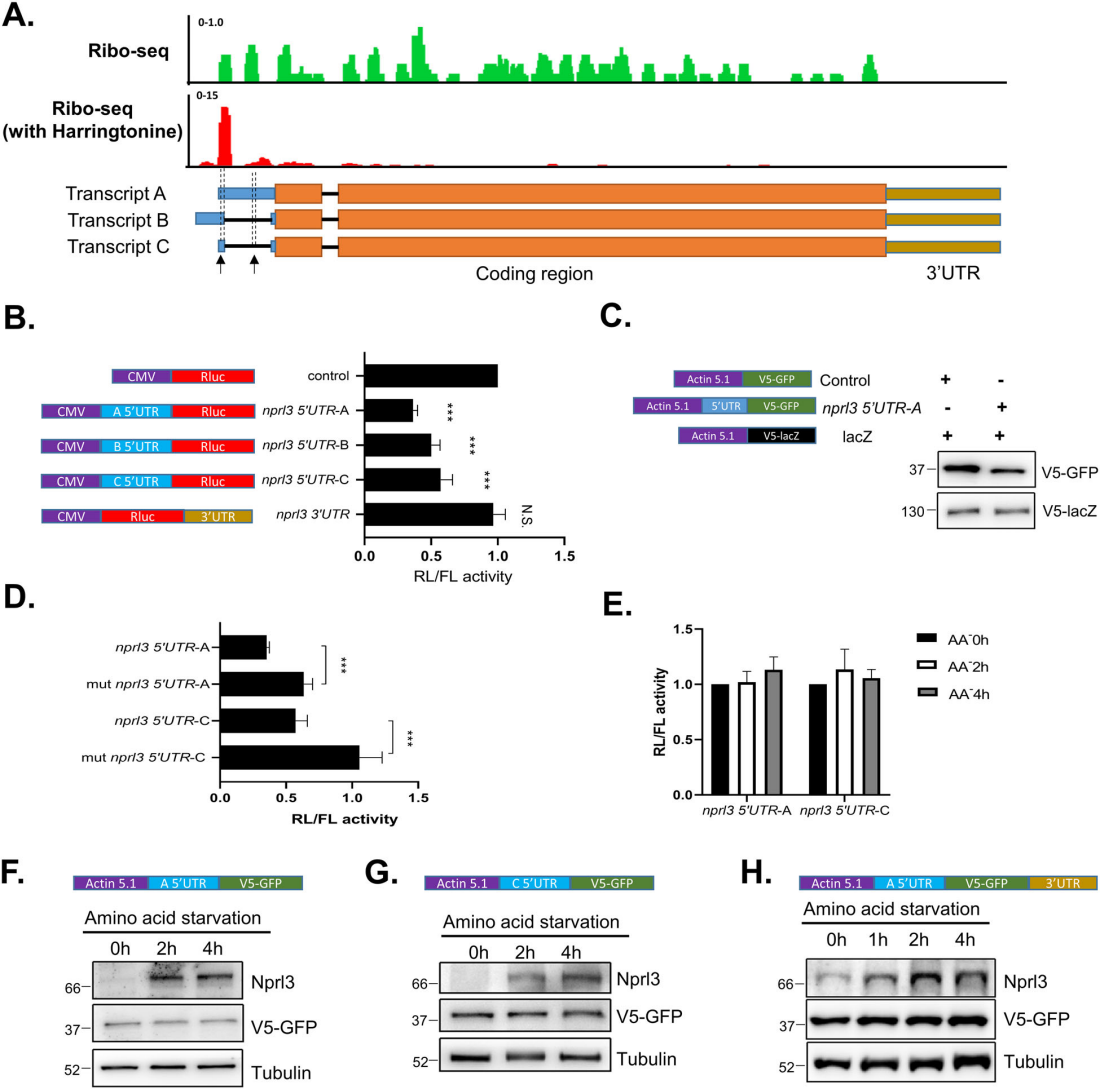
The *nprl3* 5’UTR contains functional uORFs. **A** Ribosome-protected fragment (RPF) reads mapped to the *nprl3* transcripts. The green is RPF from S2 cells without treatment, and the red is RPF from S2 cells treated with harringtonine. The three transcripts of *nprl3* are depicted. The location of the uORF that exists in all three transcripts is denoted by an arrow, and the main ORF is marked in orange. **B** S2 cells were transfected with the indicated constructs that inserted *nprl3* 5′UTR and 3′UTR to control renilla luciferase (RL) expression. The psiCHECK-2 constructs contain firefly luciferase (FL), which were used as for normalization. Values are expressed as fold change relative to the empty construct (Control). Error bars represent standard error values from three independent experiments. ***P < 0.001. **C** S2 cells were co-transfected with the V5-tagged GFP reporter construct inserted with *nprl3* 5’UTR and V5-tagged lacZ construct. The levels of V5-GFP and V5-lacZ were detected by western blot. V5-lacZ was used as a loading control for western blot. **D** S2 cells were transfected with the indicated constructs that inserted wild-type *nprl3* 5’ UTR or the mutant *nprl3* 5’UTR with the ATG in the uORF changed to GTG. Values are expressed as fold change relative to the empty construct (Control). Error bars represent standard error values from three independent experiments. ***P < 0.001. **E** S2 cells were transfected with the indicated constructs that inserted wild-type *nprl3* 5’ UTR. Cells were treated with amino acid starvation (AA−) for indicated hours. Values are expressed as fold change relative to the value before starvation. **F**–**H** S2 cells were transfected with the indicated constructs inserted with *nprl3* 5′UTR or 3′UTR to control GFP reporter expression. The cells were starved for the indicated time, and the V5-GFP was measured with western blot. α-Tubulin was used as a loading control. Similar western blot results were observed in more than three independent experiments for each group.

Eukaryotic gene expression is regulated at multiple steps, including transcription, RNA processing, mRNA translation and protein stability. To elucidate whether *nprl3* mRNA contains *cis*-elements that respond to amino acid starvation, we examined the effect of the 5′UTR and 3′UTR of the *nprl3* mRNA on translation. The *nprl3* gene encodes three transcripts that contain the same coding region and 3′UTR (Fig. 7A). The only difference between the three transcripts is 5′UTR. Interestingly, in all three mRNA isoforms, the 5′UTR sequence contains a 15 bp uORF sequence with the transcript A 5′UTR containing one additional uORF sequence (Fig. 7A). Re-analyzing the published ribosome profiling data in S2 cells^13^, we observed that while the ribosome-protected fragments (RPF) were present across the entire coding sequence (CDS) of the *nprl3* transcript, a strong peak was also present within the uORF (Fig. 7A). The highest RPF peaks around the *nprl3* uORF in the treatment of harringtonine, a translational protein synthesis inhibitor, suggested the uORF might be translated and function to inhibit downstream Nprl3 translation (Fig. 7A). To determine if the predicted uORF impacts the translation of the downstream *nprl3* coding region, we introduced the 5’UTR of the *nprl3* transcripts into the psiCHECK-2 vector in front of the *renilla luciferase* coding sequence. Intriguingly, the 5′UTR of all three *nprl3* transcripts decreased RL activity significantly, comparing to no inserted (Fig. 7B). Furthermore, placing the *nprl3* transcript A 5′UTR upstream of the GFP reporter in the pAC5.1 vector, resulted in decreased GFP protein expression (Fig. 7C). These data strongly suggest that the *nprl3* 5′UTR inhibits the translation of the downstream coding region. To validate if the ribosome-protected uORFs in 5′UTR is the *cis*-element that inhibits the downstream main ORF translation, the start codon ATG was mutated to GTG in the uORF. Critically, this mutation significantly increased the downstream reporter expression, supporting the translation inhibitory function of the uORFs (Fig. 7D).

Some functional uORFs play essential roles in modulating the translation of CDS, by decreasing their inhibition on main ORF protein translation under stress conditions^8^. To determine if nutrient depletion attenuates the inhibition of *nprl3* uORFs on downstream translation, S2 cells that ectopically expressed Renilla luciferase (RL) under the control of *nprl3* 5’UTR were starved in amino acid depletion media. Amino acid starvation did not enhance RL activity above basal levels (Fig. 7E). Consistent with this observation, amino acid starvation did not increase the levels of ectopically expressed GFP under the control of *nprl3* 5′UTR, despite the strong increase in endogenous Nprl3 protein (Fig. 7F–H). Given the RL activity under the control of transcript A 5’UTR is less than that of transcript B or C 5’UTR (Fig. 7B), the ratio of different transcripts might contribute to the Nprl3 protein expression during amino acid starvation. We detected the transcripts using two pairs of primers that generate PCR products at different sizes between transcript A and B/C. The ratio of different PCR product amounts were not significantly changed upon amino acid starvation (Supplemental Fig. 7). These findings indicate that the uORF located in the *nprl3* 5′UTR inhibits Nprl3 translation but is not sensitive to amino acid starvation.

## Discussion

**Fig. 8:**
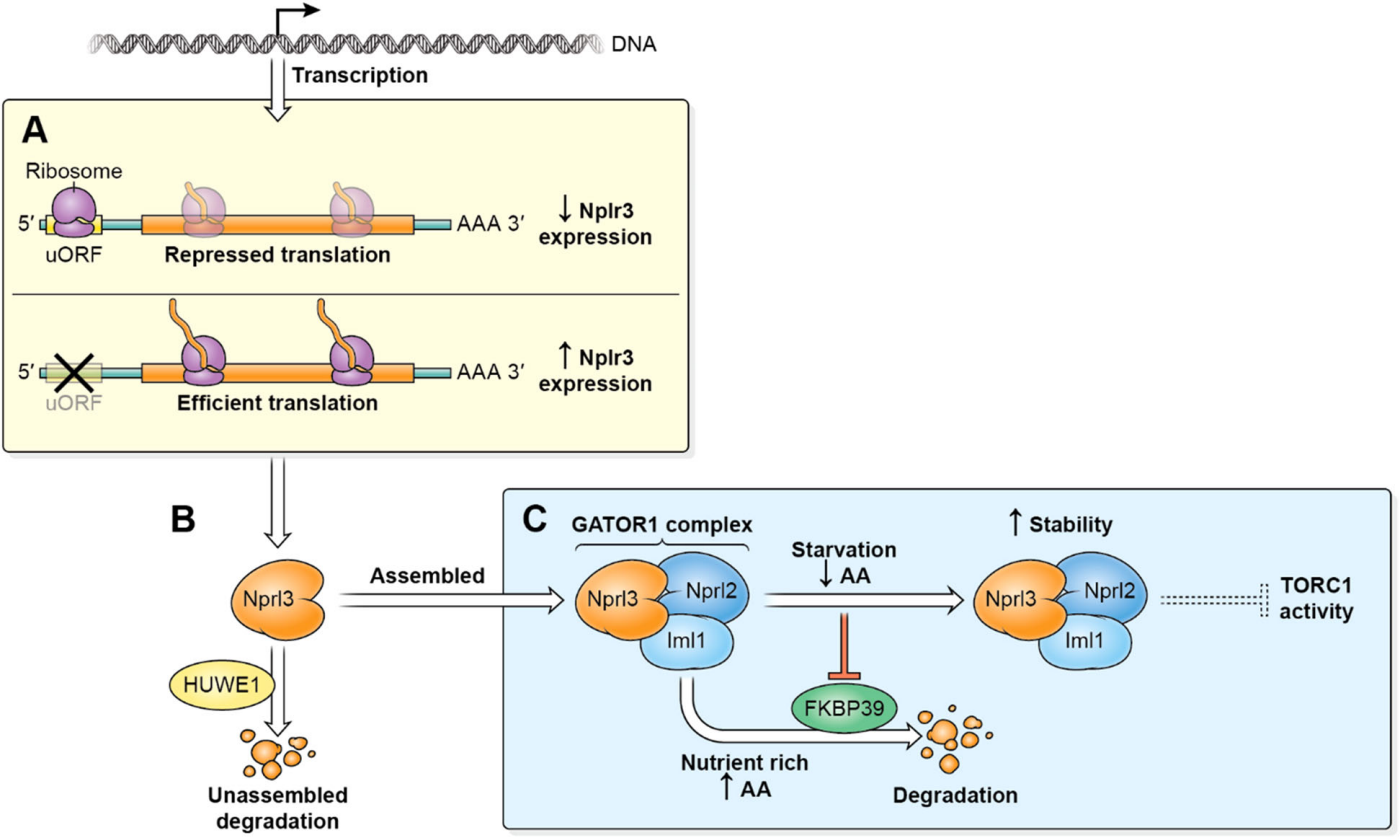
Multiple pathways regulate the levels of the TORC1 inhibitor Nprl3. **A** The *nprl3* mRNA contains a functional uORF that reduces Nprl3 translation. **B** Nprl3 forms the trimeric GATOR1 complex with the proteins Nprl2 and Iml1. When not assembled into the GATOR1 complex, Nprl3 is degraded via a HUWEI dependent pathway. **C** In nutrient-replete conditions, FKBP39 associates with Nprl3 and promotes its degradation. Upon amino acid starvation, the FKBP39 dependent destruction of Nprl3 is blocked, and the increased levels of GATOR1 resulting in decreased TORC1 activity.

The GATOR1 complex is a highly conserved inhibitor of TORC1 activity. Mutations in GATOR1 components are associated with numerous pathologies including multiple cancers and epilepsy^19,42,43^. Here we report the characterization of multiple novel pathways that regulate the expression of the essential GATOR1 component Nprl3 in Drosophila (Fig. 8). While the uORFs located at 5′UTR of *nprl3* transcripts inhibit its translation, the USPD and nutrient signaling pathways control Nprl3 protein stability. In addition, we demonstrate that the HUWE1-associated USPD pathway and FKBP39-mediated nutrient signaling independently regulate Nprl3 protein stability to control Nprl3 expression.

### FKBP39 regulates Nprl3 and Nprl2 protein stability in response to nutrient status

In metazoans, the TORC1 pathway senses multiple nutrient signals and controls protein synthesis and cell proliferation. Under amino acid depletion, the GATOR1 complex inhibits TORC1 activity and protects cell survival^31^. In mammalian cells, the GATOR1 complex component DEPDC5/Iml1 has increased protein stability under conditions of amino acid starvation while the other two GATOR1 components, Nprl2 and Nprl3, are stable and unaffected by nutrient status^26^. Here we find that in Drosophila the Nprl3 and Nprl2 proteins exhibit increased protein stability under conditions of amino acid starvation. Notably, the Drosophila genome lacks mammalian orthologues of some amino acid sensors that regulate GATOR1 activity including KICSTOR and CASTOR1^21,23^. Thus, the different responses of Nprl3 and Nprl2 expression upon starvation between Drosophila and mammals might reflect different strategies of GATOR1 regulation.

Here we find that FKBP39 interacts and promotes Nprl3 degradation under nutrient sufficient conditions. FKBP39 is the first member of the FK506-binding protein (FKBPs) family characterized in Drosophila^44^. FKBPs possess a cis-trans peptidyl-prolyl isomerase (PPIase) activity to aid protein folding as chaperons and perform some cellular functions, such as apoptosis and protein trafficking^44,45^. Interestingly, the physical interaction between FKBP39 and Nprl3 is not affected by nutrient status, which suggests that FKBP39 might be one component of a larger pathway that regulates Nprl3 stability in response to nutrient status. Notably, we also demonstrate that FKBP39 regulates the expression of another GATOR1 component Nprl2, with *fkbp39* mutants exhibiting increased levels of Nprl2 protein relative to wild type in fed conditions. Thus, in Drosophila, the FKBP39 protein functions to restrict the accumulation of both Nprl3 and Nprl2 in nutrient-replete conditions. Thus, the amino acid-sensitive FKBP39 pathway may promote the stochiometric increase in GATOR1 components upon amino acid starvation.

Consistent with a role in the regulation of TORC1, we find that mutants in *fkbp39*, which have increased levels of the TORC1 inhibitor Nprl3, have decreased TORC1 activity and increased autophagy. Thus, it is tempting to speculate that FKBP39, by preventing the accumulation of Nprl2 and Nprl3, blocks the inappropriate activation of GATOR1 and the downregulation of TORC1 activity when nutrients are plentiful. Previous results have shown that overexpression of FKBP39 inhibits fat body cell growth and autophagy during the transition from larvae to pupae in Drosophila^46^. High TORC1 activity inhibits the activation of autophagy pathways. Thus, the inhibition of autophagy in FKBP39 overexpression is consistent with our findings that FKBP39 increased TORC1 activity. However, the inhibition of cell size in FKBP39 overexpression is not consistent with our findings that FKBP39 increased TORC1 activity. Thus, FKBP39 might regulate additional pathways that impact cell size. FKBP39 is also reported to be a component of a multi-protein complex that regulates the ecdysone and juvenile hormone signal transduction pathways^47^. Whether these physiological functions of FKBP39 are associated with its roles in promoting Nprl3 degradation is an interesting question for future study.

### An upstream uORF inhibits the translation of *nprl3*

In eukaryotic genomes, the sequences of uORFs are widespread. Normally, functional uORFs inhibit their downstream main ORF. Some uORFs located in special mRNAs 5′UTR decrease their inhibition on main ORF protein translation under stress conditions^8^. One of the best-known examples is activating transcription factor 4 (ATF4), a transcriptional factor that functions in the integrated stress response^48,49^. We have determined that *nprl3* mRNA contains a functional uORF through re-analyzing ribosome profile data^13^. We demonstrate that the functional uORF downregulates the translation of the main *nprl3* ORF. However, the uORF in the *nprl3* 5′UTR is not responsive to amino acid starvation. The identification of the upstream stress or other stimuli that alter Nprl3 translation through the uORF will be an interesting area for future studies.

## Supplementary information

supplemental materials
