# Supplementary material for: FKBP39 controls nutrient dependent Nprl3 expression and TORC1 activity in Drosophila
Source: Cell Death Dis. 2021 Jun 2;12(6):571. doi: 10.1038/s41419-021-03860-z (PMC8172852; doi:10.1038/s41419-021-03860-z)
Supplement: Supplementary file 1 — supplemental materials [file 41419_2021_3860_MOESM1_ESM.docx]

**Supplemental Information**


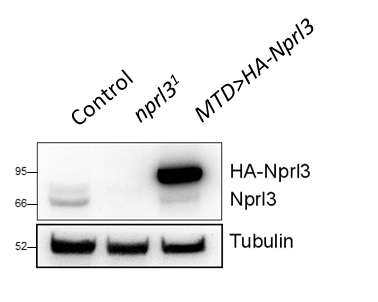


**Supplemental Figure 1.** **The anti-Nprl3 antibody can specifically recognize the Drosophila Nprl3 protein.** Western blot analysis of Nprl3 protein expression in the ovaries of *yw* (control), *nprl3^1^* and *MTD>HA-Nprl3* flies. α-Tubulin was used as a loading control.


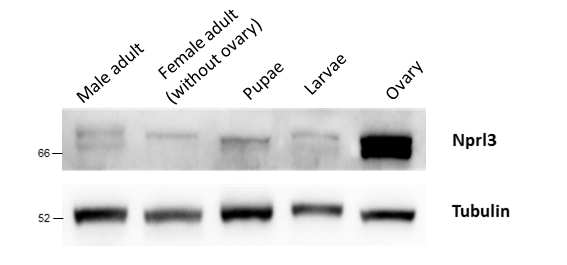


**Supplemental Figure 2.** **Expression of Nprl3 protein in Drosophila.** Western blot analysis of Nprl3 protein expression in the larvae, pupae, male adult, female adult (not including ovary) and ovaries of the *yw* flies. α-Tubulin was used as a loading control.


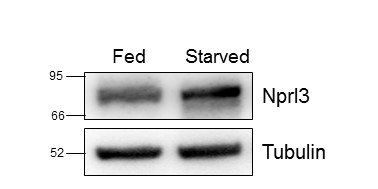


**Supplemental Figure 3. Nprl3 expression in ovaries from fed versus starved conditions.** The female flies were cultured in standard food (Fed) or starvation food (20% sucrose in PBS, Starved) for 1 day. Western blot analysis of Nprl3 protein expression in ovaries. α-Tubulin was used as a loading control.


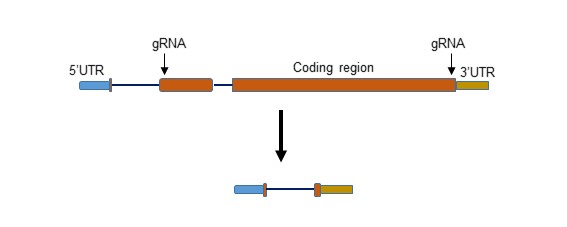


**Supplemental Figure 4. Schematic representation of *fkbp39* mutants generated using CRISPR/Cas9 genome editing.** The two guide RNA sites are indicated by arrow. Most of the *fkbp39* coding region was removed in *fkbp39^1^* mutant.


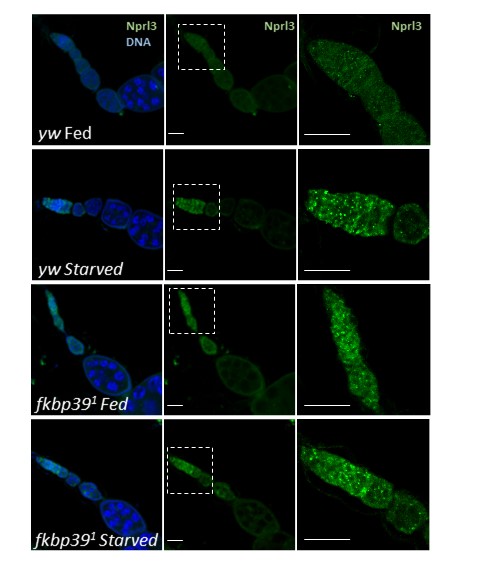


**Supplemental Figure 5.** **Nprl3 expression in wildtype and *fkbp39* mutant ovaries.** The ovaries from *yw* and *fkbp39* mutant females were cultured in complete media or amino starvation media for 2 hours and then immunostaining were done with Nprl3 antibody. The egg chambers were labeled with DAPI (blue) and Nprl3 (green). Bar, 20 μm.


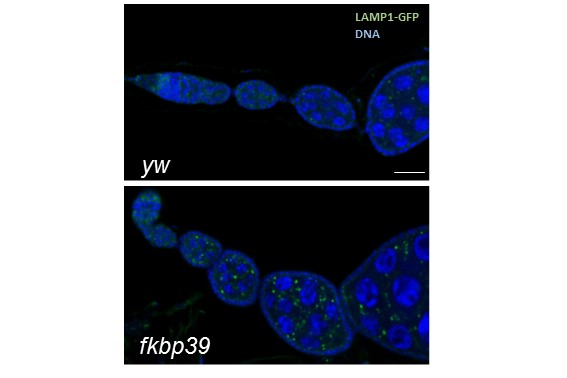


**Supplemental Figure 6. *fkbp39^1^* mutants have increased LAMP1 positive puncta.** The ovaries from *Tub-LAMP1-GFP* (control) and *Tub-LAMP1-GFP; fkbp39^1^* mutant females were labeled with DAPI (blue) and LAMP1-GFP (green). Bar, 20 μm.


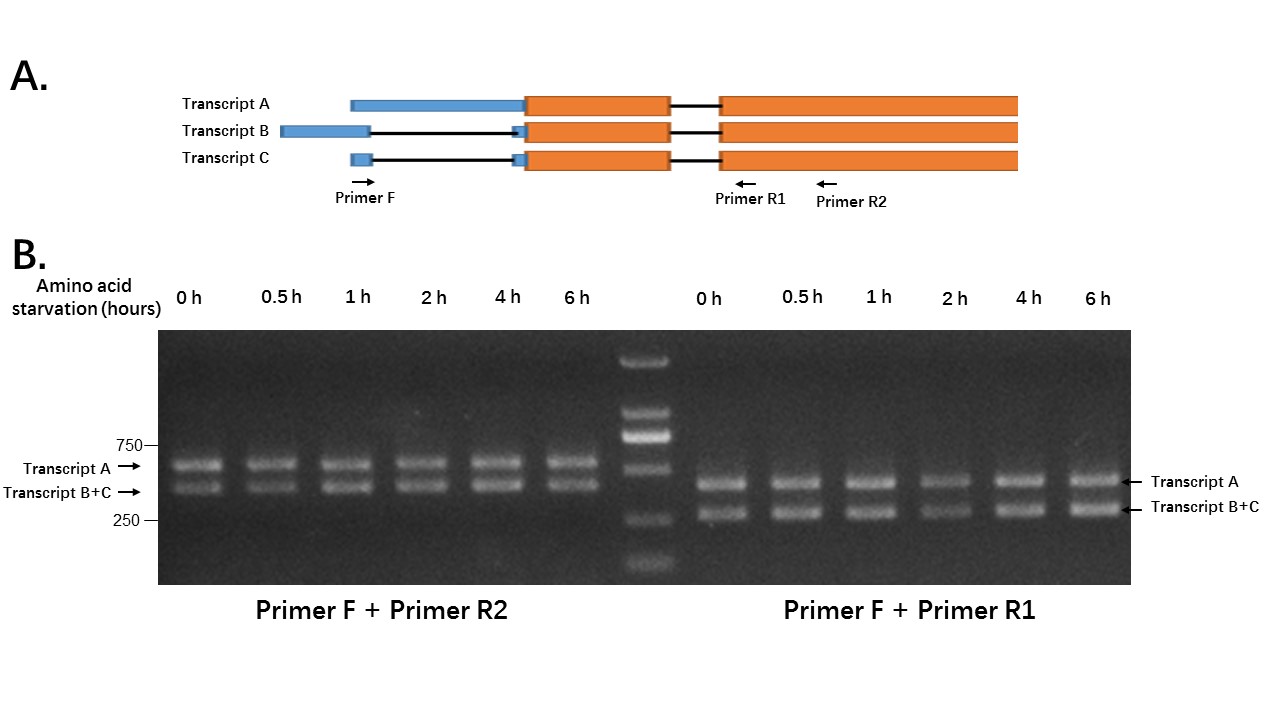


**Supplemental Figure 7.** **The amount ratio of different *nprl3* transcripts was not significantly changed upon amino acid starvation.** **A.** Schematic representation of primers used for amplifying the transcript A, B and C of *nprl3*. The 5’UTRs are marked in blue, and the coding regions are marked in orange. Two pair of primers were used to detect the ratio between A and B+C. For each pair of primer, the larger size is the product from transcript A and the smaller size is the product from transcripts B and C, because the transcript A 5’UTR contains 140 bp as intron in transcripts B and C. **B.** The S2 cells were treated with amino acid starvation media for the indicated hours, and then the RNAs were isolated from the cells. The RNAs were treated with DNase I and then used to generate cDNA. The primers were used to amplify PCR products using cDNA as template.

| Identified protein | Counts in IP from *MTD> HA-FLAG-Nprl3* | Counts in IP from control |
| --- | --- | --- |
| Act5C (negative control) | 19 | 16 |
| Nprl3 | 62 | 0 |
| Nprl2 | 15 | 0 |
| Iml1 | 22 | 0 |
| Mio | 4 | 0 |
| Seh1 | 3 | 0 |
| FKBP39 | 9 | 2 |
| HUWE1 | 4 | 0 |

**Table 1: Identification of the protein physically interacted with Nprl3 in Drosophila.**

**Table 2: primers sequence**

1. Primers used for amplifying *nprl3* 5’UTR and inserted to psiCHECK-2.

| **Primers** | **Sequence** |
| --- | --- |
| Nprl3 A 5’UTR Forward | aatacgactcactataggctagcc cttccatttctcaatgaagtcaa |
| Nprl3 A 5’UTR Reverse | tacaccttggaagccatggt ggcacacagacacttcccc |
| Nprl3 C 5’UTR Forward | aatacgactcactataggctagccctcaatgaagtcaaattgag gggaagtgtc |
| Nprl3 C 5’UTR Reverse | tacaccttggaagccatggtggcacacagacacttcccctcaatttgac |
| Nprl3 B 5’UTR Forward | aatacgactcactataggctagccgcaaactaaa agagagccac |
| Nprl3 B 5’UTR Reverse | tacaccttggaagccatggt ggcacacagacacttcccc |
| Nprl3 A 5’UTR mutant Forward | aatacgactcactataggctagcccttccatttc tcagtgaagt |
| Nprl3 B 5’UTR mutant Revers | tacaccttggaagccatggtggcacacagacacttcccctcaatttgacttcactg |
| Nprl3 C 5’UTR mutant Forward | aatacgactcactataggctagccctcagtgaag tcaaattgag gggaagtgtc |

B. Primers used for amplifying GFP and *nprl3* 5’UTR to generate pAC5.1-V5-GFP and pAC5.1-nprl3 5’UTR-V5-GFP.

| **Primers** | **Sequence** |
| --- | --- |
| GFP Forward | tagtccagtgtggtggaattcatggtgagcaagggcgagg |
| GFP Reverse | gaagggccctctagactcgagcgtggaccggtgcttgtaca |
| Nprl3 A 5’UTR Forward | gagaccccggatcggggtacccttccatttctcaatgaagtcaaatt |
| Nprl3 A 5’UTR Reverse | gcccttgctcaccatgaattcggcacacagacacttcccctt |
| Nprl3 B 5’UTR Forward | gagaccccggatcggggtaccctcaatgaagtcaaattgaggggaagtgtc |
| Nprl3 B 5’UTR Reverse | gcccttgctcaccatgaattcggcacacagacacttcccctcaatttgac |

1. Primers used for amplifying *nprl3* 5’UTR and inserted to pAC5.1-V5-Nprl3.

| **Primers** | **Sequence** |
| --- | --- |
| Npr3 A 5’UTR Forward | tggtggaattctgcagatcttccatttc tcaatgaag |
| Npr3 A 5’UTR Reverse | tcacattagtttccatgatggcacacagacacttcccc |
| Npr3 C 5’UTR Forward | tggtggaattctgcagatctcaatgaagtcaaattgaggggaagtgtc |
| Npr3 C 5’UTR Reverse | tcacattagtttccatgatggcacacagacacttcccctcaatttgac |

1. Primers used for amplifying to dsRNA template.

| **Primers** | **Sequence** |
| --- | --- |
| Fkbp39 dsRNA Forward | taatacgactcactatagggagacagaggccaagaaggaacaa |
| Fkbp39 dsRNA Reverse | taatacgactcactatagggagatgcacagctttcagttccac |
| Nprl2 dsRNA Forward | taatacgactcactatagggagatctggtctactacggggtcg |
| Nprl2 dsRNA Reverse | taatacgactcactatagggagagtacttgtggatgcagcgaa |
| Tsc1 dsRNA Forward | taatacgactcactatagggcgtaagccagccttcttcac |
| Tsc1 dsRNA Reverse | taatacgactcactatagggctccaacttgccttctttgc |
| GFP dsRNA Forward | taatacgactcactatagggagacacatgaagcagcacgactt |
| GFP dsRNA Reverse | taatacgactcactatagggagaagttcaccttgatgccgttc |
| HUWE1 dsRNA Forward | taatacgactcactatagggagacccggatctggagtggg |
| HUWE1 dsRNA Reverse | taatacgactcactatagggagaccgccgctgattccatt |

1. Primers used for qPCR detection.

| **Primers** | **Sequence** |
| --- | --- |
| nprl3 qPCR for | ACCATTCATCACTGGCCTCG |
| nprl3 qPCR rev | GATCCGCATCGCTGAAAACC |
| Rp49 qPCR for | AGATCGTGAAGAAGCGCACCAAG |
| Rp49 qPCR rev | CACCAGGAACTTCTTGAATCCGG |

1. Primers for fkbp39 CRISPR-Cas9

| **Primers** | **Sequence** |
| --- | --- |
| Fkbp39 gRNA-1 Forward | CTTCGTCGTTCCACATCTCGGGCG |
| Fkbp39 gRNA-1 Reverse | AAACCGCCCGAGATGTGGAACGAC |
| Fkbp39 gRNA-2 Forward | CTTCGTGGAGTTCGGTCCGATCTT |
| Fkbp39 gRNA-2 Reverse | AAACAAGATCGGACCGAACTCCAC |
| Fkbp39 deletion identification Forward | GAAACTCCAGTCTGAGCCCC |
| Fkbp39 deletion identification Reverse | CTCCGACCGCGCTTTTATTG |

1. Primers for detecting the ratio of *nprl3* transcript A to transcript B+C

| **Primers** | **Sequence** |
| --- | --- |
| Primer F | CTCAATGAAGTCAAATTGAG |
| Primer R1 | CGAATCCCTGCAGTTGTCCT |
| Primer R2 | GCCATTGGACCACTTCTC |
